# Characteristics and Prognosis of 8p11.23-Amplified Squamous Lung Carcinomas

**DOI:** 10.3390/jcm12051711

**Published:** 2023-02-21

**Authors:** Ioannis A. Voutsadakis

**Affiliations:** 1 Algoma District Cancer Program, Sault Area Hospital, Sault Ste. Marie, ON P6B 0A8, Canada; ivoutsadakis@nosm.ca or ivoutsadakis@yahoo.com; 2 Section of Internal Medicine, Division of Clinical Sciences, Northern Ontario School of Medicine, Sudbury, ON P3E 2C6, Canada

**Keywords:** lung cancer, squamous, NSCLC, 8p11 amplifications, NSD3, FGFR1, WHSC1L1

## Abstract

Background: Copy number alterations are common genetic lesions in cancer. In squamous non-small cell lung carcinomas, the most common copy-number-altered loci are at chromosomes 3q26-27 and 8p11.23. The genes that may be drivers in squamous lung cancers with 8p11.23 amplifications are unclear. Methods: Data pertaining to copy number alterations, mRNA expression and protein expression of genes located in the 8p11.23 amplified region were extracted from various sources including The Cancer Genome Atlas, the Human Protein Atlas and the Kaplan Meier Plotter. Genomic data were analyzed using the cBioportal platform. Survival analysis of cases with amplifications compared to nonamplified cases was performed using the Kaplan Meier Plotter platform. Results: The 8p11.23 locus is amplified in 11.5% to 17.7% of squamous lung carcinomas. The most frequently amplified genes include *NSD3*, *FGFR1* and *LETM2*. Only some of the amplified genes present concomitant overexpression at the mRNA level. These include *NSD3*, *PLPP5*, *DDHD2*, *LSM1* and *ASH2L*, while other genes display lower levels of correlation, and still, some genes in the locus show no mRNA overexpression compared with copy-neutral samples. The protein products of most locus genes are expressed in squamous lung cancers. No significant difference in overall survival in 8p11.23-amplified squamous cell lung cancers versus nonamplified cancers is observed. In addition, there is no adverse effect of mRNA overexpression for relapse-free survival of any of the amplified genes. Conclusion: Several genes that are part of the commonly amplified locus 8p11.23 in squamous lung carcinomas are putative oncogenic candidates. A subset of genes of the centromeric part of the locus, which is amplified more commonly than the telomeric part, show high concomitant mRNA expression.

## 1. Introduction

Lung cancer is the most prevalent cancer worldwide and the leading cause of cancer death [1]. Two main types of lung cancers are distinguished histologically: non-small cell lung cancers (NSCLCs), which are the most common, and small cell lung cancers (SCLCs), which represent about 15% to 20% of the total lung cancers. NSCLC is divided into two main subtypes, adenocarcinomas and squamous lung cancers, which have distinct molecular pathogenesis. Based on molecular abnormalities, some adenocarcinomas are currently treated with targeted therapies against EGFR, ALK or ROS kinases [2]. Immunotherapies inhibiting CTLA-4 or the PD-L1/PD-1 ligand/receptor pair are also effective for subsets of patients with both subtypes of NSCLC [3,4]. Besides immunotherapies, no other targeted therapies currently exist for squamous cell lung cancers. Thus, there is a need for further rationally developed targeted therapies for this type of lung cancer based on molecular defects. The molecular landscape of squamous cell lung cancer has been elucidated by TCGA and consists of recurrent mutations in 11 genes and a mean of 360 exonic mutations per tumor as well as a mean of 323 copy number alterations [5]. Copy number alterations (CNAs), both gains and losses, are common molecular lesions in cancer and complement mutations and epigenetic changes in neoplastic pathogenesis [6]. Recurrent CNAs may be promoted by increasing the expression of oncogenes or leading to tumor suppressor losses. However, often, the gained or lost region contains several genes and most of them are passenger alterations with no pathophysiologic benefit to the cancer cell. In many cases of recurrent CNAs, the possible driver oncogene(s) or tumor suppressor(s) is not well defined.

In squamous NSCLC, the most commonly recurrently amplified area, in about 40% of cases, is at chromosome locus 3q26-27, which includes the oncogene SOX2, a transcription factor and member of a panel of factors that are able to reprogram differentiated cells to pluripotent stem cells [7]. SOX2 is involved in lung organogenesis and squamous differentiation [8]. Other oncogenes located in this locus include PIK3CA and MECOM, encoding for EVI1. Another amplified area in squamous NSCLC is at chromosome 8p11.23, which is the focus of this report. This locus is amplified in about one in six squamous NSCLCs and has been also reported to be amplified in breast cancers and urothelial bladder carcinomas [9,10,11].

## 2. Methods

Publicly available genomic data pertaining to the squamous subtype of NSCLC from The Cancer Genome Atlas (TCGA) were extracted using the cBioCancer Genomics Portal (cBioportal, http://www.cbioportal.org, last accessed: 12 November 2022), a site that allows for interrogation of data for genetic alterations such as mutations and CNAs as well as mRNA expression of any gene of interest [5,12]. CNAs are computed in TCGA using the Genomic Identification of Significant Targets in Cancer (GISTIC) algorithm. The algorithm assigns a putative amplification status to genes with a score of 2 or higher. A global aneuploidy status of each case is also provided in TCGA by an aneuploidy score (AS) representing the sum of chromosomal arms with gains or losses in Affymetrix 6.0 SNP arrays. The definition of a CNA of a whole chromosome arm for the calculation of AS consists of somatic copy number alterations in more than 80% of the total arm length as determined by the ABSOLUTE algorithm [13]. In contrast, chromosomal arms with alterations in less than 20% of their total length are defined as not copy-number-altered, while chromosome arms with alterations involving 20% to 80% of their length are not called, and thus considered not altered, according to the algorithm. The RSEM algorithm was used for normalization of mRNA expression [14].

The Human Protein Atlas (www.proteinatlas.org, last accessed: 19 November 2022), a publicly available database of protein expressions in human normal and neoplastic tissues, was interrogated for the expression of proteins of genes located at chromosome location 8p11.23 in squamous lung cancer [15]. The Human Protein Atlas contains data from a semi-quantitative immunohistochemistry-based evaluation of the expression of proteins in human tissues. On many occasions, evaluations have been carried out with several different commercially available antibodies for each protein.

The overall survival (OS) of 8p11.23-amplified squamous lung cancers (defined as NSD3 amplified per the GISTIC algorithm definition) and nonamplified squamous lung cancers was determined in the TCGA cohort. The prognosis of squamous lung cancer patients according to the mRNA expression level of each of the 8p11.23 genes and association with relapse-free survival (RFS) was tested using data from a series contained in the online publicly available platform Kaplan Meier Plotter (www.kmplot.com, last accessed: 18 November 2022) [16]. The cut-off of amplified and nonamplified samples for each gene was set at the higher quartile of amplification, as the closer cut-off to the percentage of lung cancer cases with 8p11.23 locus amplifications, provided by the kmplot platform.

Statistical comparisons of categorical and continuous data were carried out with Fisher’s exact test or the χ^2^ test and the *t* test. The log-rank test was used to compare Kaplan–Meier survival curves. All statistical comparisons were considered significant if *p* < 0.05 except for the RFS survival analysis according to mRNA expression levels of different genes, which was considered significant at a *p* < 0.0005 level to account for multiple comparisons.

## 3. Results

**Figure 1:**
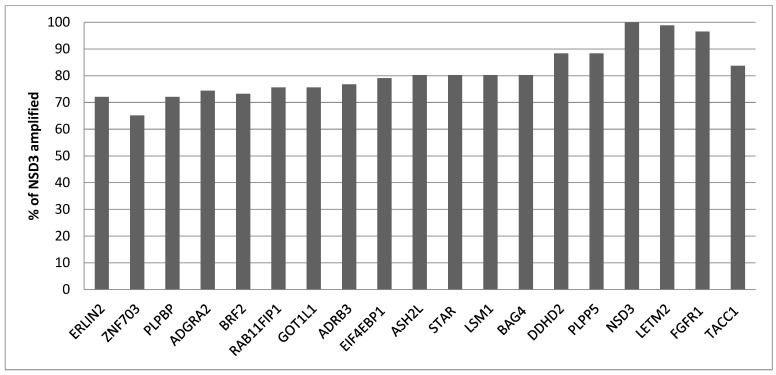
Percentage of amplification of each of the other 8p11.23 genes in samples with NSD3 amplification in TCGA squamous lung cancer study. TCGA: The Cancer Genome Atlas.

**Figure 2:**
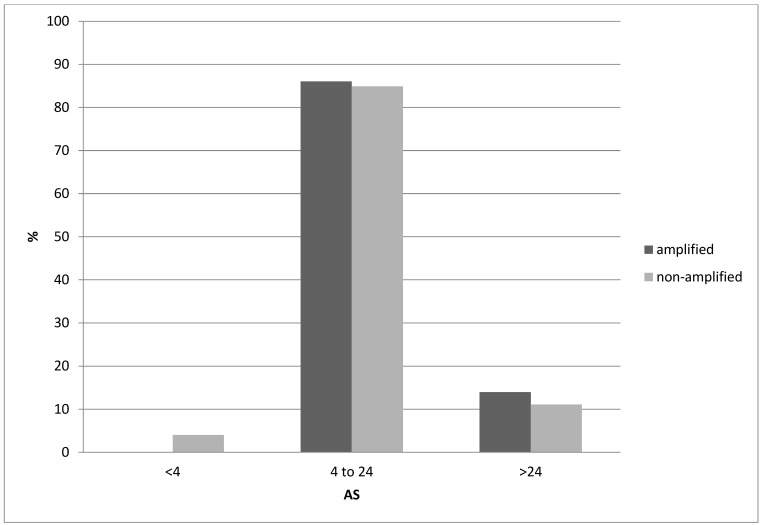
Percentage of cases with the 8p11.23 amplicon and without the amplicon and aneuploidy score (AS) of less than 4, between 4 and 24 and above 24. Data are from TCGA lung squamous carcinoma study. TCGA: The Cancer Genome Atlas.

**Figure 3:**
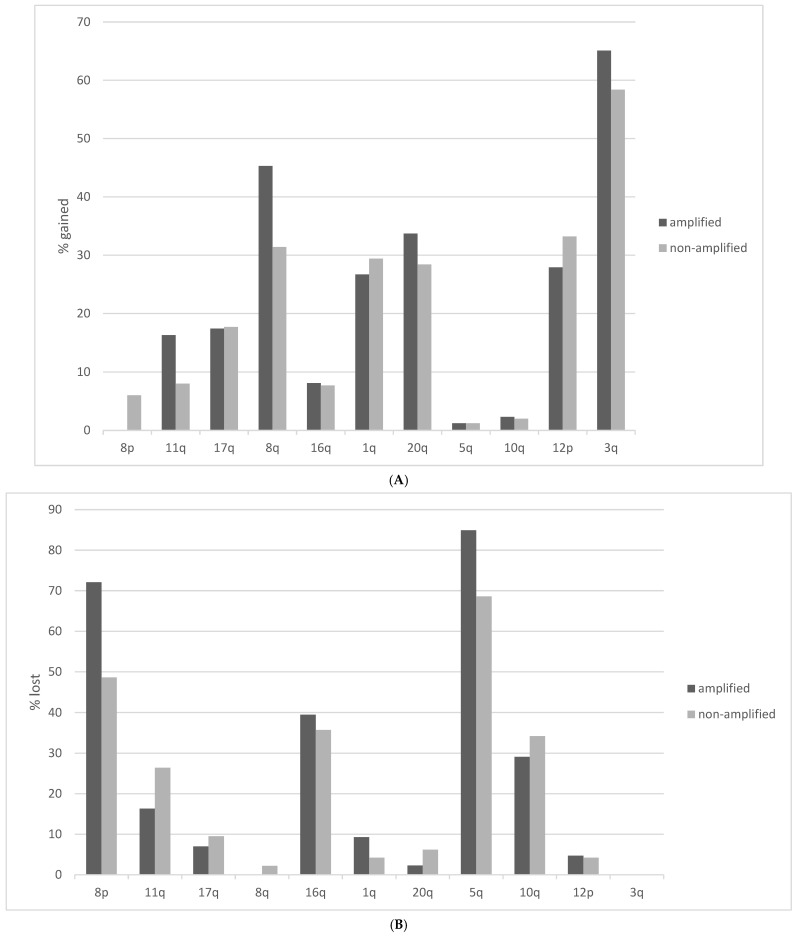
Percentage of chromosome arm gains or losses in squamous lung cancer samples with the 8p11.23 amplicon and without the amplicon in TCGA study. (**A**) Gains. (**B**) Losses. TCGA: The Cancer Genome Atlas.

Squamous lung cancers with amplification of the 8p11.23 locus, as defined by *NSD3* amplification, do not differ from non-8p11.23-amplified carcinomas in the mean age of patients at presentation, the percentage of patients older than 65 years old or in the sex and race distribution (Table 1). The stage at diagnosis is also similar between the two groups. Genes located at the 8p11.23 locus are amplified in 11.5% to 17.7% of squamous NSCLCs (Table 2). A higher frequency of amplification is observed in the most centromeric parts of the locus with genes *NSD3*, *FGFR1* and *LETM2* showing a higher number of amplified cases in TCGA (Figure 1). Genes in the central and more telomeric parts of the locus display progressively lower frequencies of amplification, with the most telomeric genes ERLIN2 and ZNF703 being amplified in 60% to 70% of NSD3-amplified cases (Figure 1). Conversely, in NSD3 nonamplified cases, the rest of the genes of the locus are amplified only rarely, in isolated cases (not shown). The global copy number alteration burden of 8p11.23-amplified and nonamplified squamous NSCLC as measured by the AS was not different, with most cases in both groups having an intermediate AS between 4 and 24 (Figure 2). No cases in the amplified group had an AS below 4, but even in the nonamplified group, only 4% of cases had an AS below 4. The mean AS of the amplified group was 16.29 (SD: 6.45), and the mean AS of the nonamplified group was 16.08 (SD: 6.64, unpaired *t* test *p* = 0.78). Individual chromosome arms with more frequent gains in the 8p11.23-amplified group compared to the nonamplified group included 8q (45.3% in the 8p11.23-amplified group versus 31.4% in the nonamplified group, *p* = 0.01) and 11q (16.3% versus 8%, respectively, *p* = 0.02) (Figure 3A). In contrast, arm 8p showed no gains in any of the cases in the 8p11.23-amplified group and it was gained in 6% of the nonamplified group. Chromosome arms with losses occurring more frequently in 8p11.23-amplified squamous NSCLC included 8p (72.1% versus 48.6% in the nonamplified group, *p* = 0.0001) and 5q (84.9% versus 68.6% in the nonamplified group, *p* = 0.003) (Figure 3B). Chromosome arm 11q was more frequently (but not statistically significantly) lost in the 8p11.23 nonamplified group (26.4% versus 16.3% in the amplified group, *p* = 0.06).

**Figure 4:**
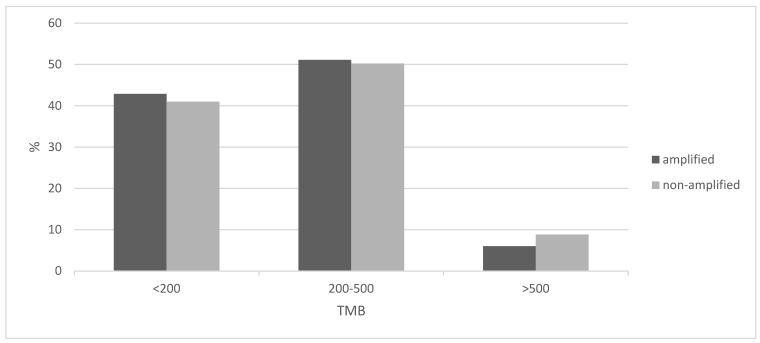
Percentage of cases with the 8p11.23 amplicon and without the amplicon and tumor mutation burden (TMB) of different levels. Data are from TCGA lung squamous carcinoma study. TCGA: The Cancer Genome Atlas.

**Figure 5:**
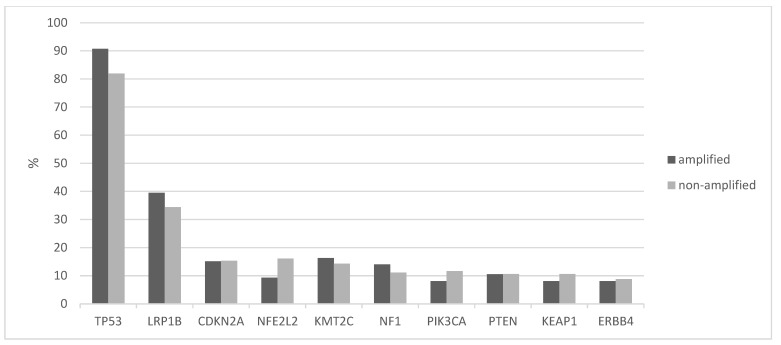
Comparison of percentage of mutations in commonly mutated genes between the 8p11.23-amplified and nonamplified groups in the squamous cell carcinoma samples of TCGA study. TCGA: The Cancer Genome Atlas.

The tumor mutation burden (TMB) was similar in 8p11.23-amplified and nonamplified squamous NSCLC, with about half of the patients in both groups having a TMB between 200 and 500 mutations and about 5% of the amplified group and a slightly higher percentage of the nonamplified group presenting a TMB above 500 (Figure 4). The frequency of the two categories with mutation numbers above 200, which display a higher probability of responses to immunotherapy with immune checkpoint inhibitors, did not differ significantly between the 8p11.23-amplified and nonamplified groups (Fisher’s exact test *p* = 0.9). The mean mutation number of the 8p11.23-amplified group was 271.9 (SD: 152.8) and did not differ from the mean mutation number of the nonamplified group, which was 270.9 (SD: 199.6, unpaired *t* test *p* = 0.9). Among individual oncogene mutations, *TP53* mutations were more common in the 8p11.23-amplified group (90.7% versus 81.9% in the nonamplified group, Fisher’s exact test *p* = 0.05), while the master hypoxia response transcription factor NFE2L2 was more often mutated in the nonamplified group but not statistically significant (16.1% versus 9.3% in the 8p11.23-amplified group, Fisher’s exact test *p* = 0.13). The prevalence of PIK3CA mutations in the amplified group (8.1%) was also not different from the nonamplified group (11.6%, Fisher’s exact test *p* = 0.44). Figure 5 shows the percentage of mutations in the most frequently mutated oncogenes in squamous NSCLC in the two groups.

**Figure 6:**
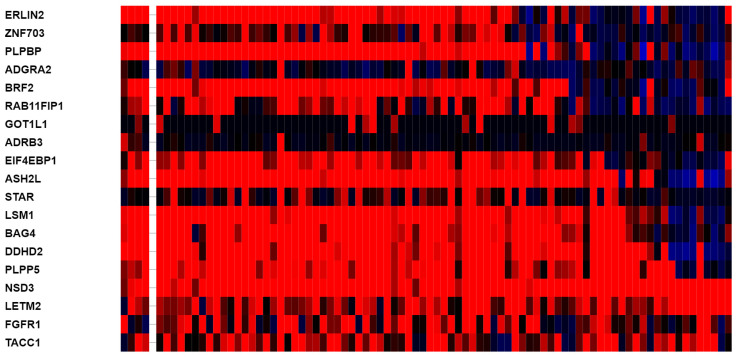
mRNA expression (z score compared with diploid samples) of genes at 8p11.23 in 8p11.23-amplified cases in squamous lung cancer samples of TCGA cohort. Red denotes overexpression and blue denotes suppressed expression.

Another chromosome area that is most frequently amplified in squamous NSCLC is 3q26, which harbors oncogenes *SOX2*, *PIK3CA* and *MECOM*. An evaluation of 8p11.23-amplified and nonamplified samples disclosed that *SOX2*, *PIK3CA* and *MECOM* genes are coamplified in similar percentages of cases (40.7% versus 39.7% for *SOX2*, 38.4% versus 35.2% for *MECOM* and 38.4% versus 37.7% for *PIK3CA,* respectively). Increased mRNA expression of the amplicon genes in amplified cases correlates with gene amplification in several genes but not in others. The most frequently amplified gene of the amplicon *NSD3* shows higher mRNA overexpression in over 90% of the amplified samples. In contrast, the two neighboring genes *FGFR1* and *LETM2*, which are almost invariably coamplified, are overexpressed at the mRNA level in about half or less of the amplified cases (Figure 6). Four other genes that show high mRNA expression (over 70% of amplified cases with a z score above 2 compared with diploid samples) include *PLPP5*, *DDHD2*, *LSM1* and *ASH2L*. Four genes, *ADGRA2*, *GOT1L1*, *ADRB3* and *STAR*, are not overexpressed in any amplified cases. Besides *ADGRA2*, *ADRB3* and *STAR*, which are not expressed, protein products of genes of 8p11.23 are in general expressed in squamous NSCLC, at least with one antibody checked (Table 3). However, there was significant variability depending on the antibody used.

**Figure 7:**
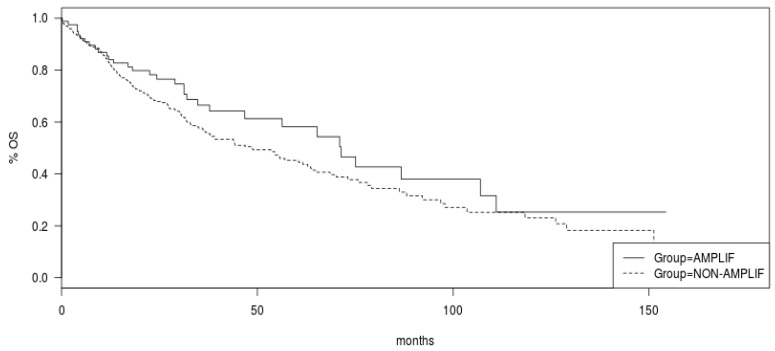
Overall survival of 8p11.23-amplified cases of squamous lung cancer samples versus nonamplified counterparts. Log-rank *p* = 0.12. Data are from TCGA. AMPLIF: 8p11.23-amplified group, NON-AMPLIF: 8p11.23 nonamplified group.

Although the OS of the 8p11.23-amplified squamous lung carcinoma group in TCGA was better than the OS of the nonamplified group, this difference did not reach statistical significance (log-rank *p* = 0.12, Figure 7). Relapse-free survival (RFS) of squamous NSCLC patients in the higher quartile of mRNA expression of NSD3 was no different from counterparts in the three lower quartiles (not shown). Surprisingly, the RFS of patients in the higher quartile of mRNA expression of FGFR1 expression was improved compared with patients in the three lower quartiles (not shown). All other genes besides PLPP5, which showed better RFS in the mRNA overexpressed group, showed no significant differences in RFS between groups (not shown).

## 4. Discussion

Chromosomal locus 8p11.23 is the second most frequently amplified locus in the squamous histology of lung cancers, and squamous NSCLC is a type of cancer with a higher frequency of amplifications in this locus among all cancer histologies and primary locations. In contrast, adenocarcinomas of the lung display amplifications in this chromosomal locus at a lower frequency (about 2.5% to 3% of cases in TCGA lung adenocarcinoma study). Genes located at 8p11.23 include receptor tyrosine kinase FGFR1; two methyl-transferases, ASH2L, which is part of the mixed lineage leukemia (MLL) complex, and NSD3; two phospholipid phosphatases, DDHD2 and PLPP5; and proteins ZNF703 and BRF2, which are transcription regulators (Table 1). Two proteins, EIF4EBP1 and LSM1, located at 8p11.23 are involved in mRNA translation and metabolism. A list of additional genes amplified in squamous NSCLC is shown in Table 1. Previous studies have examined the implications of some 8p11.23 genes in lung cancer. ZNF703 is a transcription factor with roles in development and in ER-positive breast cancers where it is associated with more aggressive subsets [17]. In lung cancer, samples with ZNF703 amplification displayed variable mRNA overexpression, suggesting an imperfect correlation [18]. Another transcription regulator from 8p11.23, BRF2, is a subunit of transcription factor TFIIIB [19]. TFIIIB co-operates in transcription guided by RNA polymerase III, the polymerase transcribing tRNA genes. Thus, BRF2 plays an important role in the regulation of protein synthesis, with implications for proliferating cancer cells. In lung cancer, pathways upregulating BRF2 have been shown to favor cancer progression [20,21]. BAG4, a protein transcribed from a gene at 8p11.23 with a role in the inhibition of apoptosis, has been shown to transform breast cells and may have functional implications for lung cancer, being commonly coamplified with FGFR1 and NSD3 [22,23]. The current study examines the 8p11.23-amplified area and the nineteen genes that are located at this chromosomal locus in squamous NSCLC. The analysis based on published genomic data shows that the genes of the locus are amplified en bloc in the majority of amplified cases, while in fewer cases, only a subset of genes at 8p11.23 are amplified. The higher frequency of amplification among the genes of the 8p11.23 locus is observed in the most centromeric genes including *NSD3*, *LETM2* and *FGFR1*. The amplification of 8p11.23 has no significant influence on the TMB or the AS of the cases, suggesting that genes in the locus are not involved in aneuploidy or DNA repair mechanisms of the cancer cells. In addition, no influence of 8p11.23 amplification on the prevalence of the other frequent CNA in squamous NSCLC, the amplification of 3q26 is observed. mRNA expression of the amplified genes is variable, with a higher correlation of amplification and overexpression observed in several genes in the most centromeric part of the locus (*NSD3*, *PLPP5*, *DDHD2, LSM1* and *ASH2L*) and also in a few genes that are located toward the telomeric end of the area (*BRF2* and *PLPBP*). Several other genes show lower or no increase in mRNA expression in amplified cases. Interestingly, increased mRNA expression was not associated with worse patient RFS for any of the genes amplified at 8p11.23. The caveat of this survival analysis is that, in the increased-mRNA-expression group, up to one-third of patients may be nonamplified, as the cut-off was at the highest quartile of expression. These data suggest that genes amplified at 8p11.23 do not confer survival or other cancer-related process benefit in squamous NSCLC but may be amplified as part of an underlying defect that makes the locus prone to repeated DNA replication. However, given the limitations of the survival analysis, it cannot be totally excluded that a survival benefit of increased copy numbers of one or more 8p11.23 genes exists for squamous NSCLC cells. The centromeric area of the 8p11.23 amplicon that contains genes *NSD3*, *LETM2*, *FGFR1* and *TACC1* and presents a higher amplification frequency in squamous NSCLC is homologous to an area at human chromosome 4p that contains genes related to each of the 8p11 genes. These include *NSD2*, *LETM1*, *FGFR3* and *TACC3*. This 4p area, although rarely amplified, it is the site of fusions between *FGFR3* and *TACC3* in a small percentage (1.2%) of squamous lung carcinoma cases, while *FGFR1* and *FGFR2* fusions are observed in isolated cases [5]. In bladder cancer, which is also a type of cancer that harbors 8p11.23 amplifications, the same fusions are present in the homologous 4p location. In addition, bladder cancers present mutations of *FGFR3*, which make them susceptible to treatment with FGFR inhibitors [24]. The presence of genetic abnormalities in these homologous regions harboring genes with similar functions in the same cancers would argue for a functional benefit conferred by one or more genes. The prime candidate in this case is obviously FGFR family members for which clinical data for the efficacy of their inhibition in other cancers exist, implying functional dependence of cancers carrying FGFR genetic lesions, at least in some cases. Related to FGFR1 targeting in 8p11.23-amplified squamous lung carcinomas, it is worth noting that, as shown here, amplification is not always associated with mRNA overexpression. This is important when considering FGFR1 amplifications as potential biomarkers for the development of treatments with FGFR inhibitors or inhibitors of the downstream cascades such as PI3K inhibitors [25,26].

An improved understanding of recurrent copy number alterations in cancer and the genes that are affected by them as well as the characterization of candidate driver genes in altered areas present therapeutic opportunities. The proof of principle has already been provided decades ago when amplifications of 17q in a subset of breast cancer genes were revealed to harbor ERBB2 amplifications and became the basis for effective therapies that have changed the outcomes of HER2-positive breast cancers [27,28]. A similar opportunity may arise with 8p11.23 amplifications and the development of FGFR inhibitors. Clinical translation will require a better grasp of driver genes in the locus and clarification of additional drivers. Methyltransferase NSD3 is a serious candidate and it has been shown to promote squamous lung carcinoma proliferation in experimental models in vitro and in vivo by promoting the transcription of oncogenes through lysine methylation of histone 3 at position 36 [29,30]. Pathways activated by driver genes creating tumor dependency would be important to elucidate. For FGFR receptors, for example, transactivation of additional tyrosine kinase receptors may be at play and could have implications for inhibition effectiveness in cancers with defects in these other tyrosine kinase receptors such as HER2 amplifications [31]. The development of companion diagnostics for the measurement of the amplification with the most effective cut-off for clinical efficacy will also be of paramount importance as the case of HER2 has shown.

## Figures and Tables

**Table 1:** Characteristics of the groups with and without 8p11.23 amplifications in patients with squamous lung carcinomas from the squamous lung cancer TCGA study. TCGA: The Cancer Genome Atlas, SD: Standard Deviation, NA: Not Available.

Characteristic	Whole Group (*n* = 487) (%)	8p11.23 Amplified (*n* = 86) (%)	8p11.23 Nonamplified (*n*= 401) (%)	*p*
Age at diagnosis (mean ± SD)	67.3 ± 8.5	67.3 ± 7.7	67.3 ± 8.7	1.0
Age				
≤65 years old	182 (37.4)	31 (36)	151 (37.6)	0.8
>65 years old	294 (60.4)	53 (61.7)	241 (60.1)	
NA	11 (2.2)	2 (2.3)	9 (2.3)	
Sex				
Male	358 (73.5)	68 (79.1)	290 (72.3)	0.27
Female	127 (26.1)	18 (20.9)	109 (27.2)	
NA	2 (0.4)		2 (0.5)	
Race				
White	337 (69.2)	56 (65.1)	281 (70.1)	0.84
Black	29 (6)	4 (4.7)	25 (6.2)	
Asian	9 (1.8)	1 (1.2)	8 (2)	
NA	112 (23)	25 (29.1)	87 (21.7)	
Stage at diagnosis				
I	236 (48.5)	37 (43)	199 (49.7)	0.53
II	158 (32.4)	29 (33.7)	129 (32.2)	
III	83 (17)	18 (21)	65 (16.1)	
IV	7 (1.4)	2 (2.3)	5 (1.2)	
NA	3 (0.6)		3 (0.7)	

**Table 2:** Genes at the 8p11.23 locus. Positions are according to human genome version GRCh38. The last column presents the number of samples in TCGA squamous lung cancer study with amplifications in each gene with percentage in parentheses.

Official Name	Alternative Names	Position	Lung Squamous Carcinoma, *n* = 487 (%)
ERLIN2 (Endoplasmic Reticulum Lipid Raft Associated 2)	SPFH2	37,736,601–37,758,422	62 (12.7)
ZNF703 (Zinc Finger Protein 703)	FLJ14299, ZEPPO1	37,695,782–37,700,019	56 (11.5)
PLPBP (Pyridoxal phosphate binding protein)	PROSC	37,762,595–37,779,768	62 (12.7)
ADGRA2 (Adhesion G protein coupled receptor A2)	GPR124	37,784,191–37,844,896	64 (13.1)
BRF2 (RNA polymerase III transcription initiation factor subunit)	TFIIIB50	37,843,268–37,849,861	63 (12.9)
RAB11FIP1 (RAB11 Family Interacting Protein 1)		37,858,618–37,899,497	65 (13.3)
GOT1L1 (Glutamic-oxaloacetic transaminase 1-like 1)	MGC33309	37,934,281–37,940,124	65 (13.3)
ADRB3 (Adrenoreceptor beta 3)		37,962,990–37,966,599	66 (13.6)
EIF4EBP1 (Eukaryotic transcription initiation factor 4E binding protein 1)	4E-BP1	38,030,534–38,060,365	68 (14)
ASH2L (ASH2-like histone lysine methyltransferase complex subunit)		38,105,493–38,144,076	69 (14.2)
STAR (Steroidogenic acute regulatory protein)	STARD1	38,142,700–38,150,992	69 (14.2)
LSM1 (LSM1 homolog, mRNA-degradation-associated)	CASM	38,163,335–38,176,730	69 (14.2)
BAG4 (BAG cochaperone 4)	SODD	38,176,533–38,213,301	69 (14.2)
DDHD2 (DDHD domain containing 2)		38,225,218–38,275,558	76 (15.6)
PLPP5 (Phospholipid phosphatase 5)	PPAPDC1B, HTPAP	38,263,130–38,269,243	76 (15.6)
NSD3 (Nuclear receptor binding SET domain protein 3)	WHSC1L1	38,269,704–38,382,272	86 (17.7)
LETM2 (Leucine zipper and EF-hand containing transmembrane protein 2)	SLC55A2	38,386,207–38,409,527	85 (17.5)
FGFR1 (Fibroblast Growth Factor Receptor 1)	CD331	38,400,215–38,468,834	83 (17)
TACC1 (Transforming Acidic Coiled Coil Containing protein 1)		38,728,186–38,853,028	72 (14.8)

**Table 3:** Protein expression by immunohistochemistry (IHC) of 8p11.23 proteins in squamous lung carcinomas from the Human Protein Atlas. In IHC staining columns, intensity of staining has been grouped as none/low and medium/high. The second column shows the antibody commercial catalogue number, type and company. r pAb: rabbit polyclonal antibody, r mAb: rabbit monoclonal antibody, m mAb: mouse monoclonal antibody, NA: not available.

Official Name	Primary Antibody	IHC Staining (Number of Samples)	
		None–Low	Medium–High
ERLIN2	HPA002025 (r pAb) Sigma-Aldrich	4	8
	CAB014894 (r mAb) Origene	0	11
ZNF703	HPA023930 (r pAb) Sigma-Aldrich	10	1
	CAB068249 (m mAb) Sigma-Aldrich	0	12
PLPBP	HPA023646 (r pAb) Sigma-Aldrich	11	1
	HPA023733 (r pAb) Sigma-Aldrich	11	1
	CAB017033 (m mAb) Origene	4	5
ADGRA2	HPA012393 (r pAb) Sigma-Aldrich	12	0
BRF2	HPA023378 (r pAb) Sigma-Aldrich	0	11
	CAB019269 (r mAb) Origene	2	9
RAB11FIP1	HPA023904 (r pAb) Sigma-Aldrich	3	8
	HPA024010 (r pAb) Sigma-Aldrich	9	1
	HPA025960 (r pAb) Sigma-Aldrich	2	9
	CAB017037 (r mAb) Origene	5	5
GOT1L1	HPA028778 (r pAb) Sigma-Aldrich	7	4
ADRB3	HPA061969 (r pAb) Sigma-Aldrich	12	0
EIF4EBP1	HPA023501 (r pAb) Sigma-Aldrich	4	7
	CAB005032 (r mAb) Epitomics	3	8
	CAB005039 (r mAb) Epitomics	8	2
ASH2L	HPA042289 (r pAb) Sigma-Aldrich	3	7
STAR	HPA023644 (r pAb) Sigma-Aldrich	11	0
	HPA027318 (r pAb) Sigma-Aldrich	11	0
	CAB032598 (r pAb) Santa Cruz Biotechnology	11	0
LSM1	NA		
BAG4	HPA018951 (r pAb) Sigma-Aldrich	3	7
	CAB013716 (r mAb) Origene	1	11
DDHD2	HPA023143 (r pAb) Sigma-Aldrich	9	1
	HPA023147 (r pAb) Sigma-Aldrich	8	3
	CAB015202 (r mAb) Origene	4	8
PLPP5	NA		
NSD3	HPA005659 (r pAb) Sigma-Aldrich	5	7
	HPA018893 (r pAb) Sigma-Aldrich	0	10
	CAB013721 (r mAb) Origene	0	10
LETM2	HPA025032 (r pAb) Sigma-Aldrich	11	1
FGFR1	HPA056402 (r pAb) Sigma-Aldrich	12	0
	CAB033614 (m mAb) Santa Cruz Biotechnology	5	5
TACC1	HPA024702 (r pAb) Sigma-Aldrich	11	1
	CAB017041 (r mAb) Origene	2	7

## Data Availability

No data beyond those included in the manuscript are available.
